# Chemotherapy Administration during Pelvic Radiation for Cervical Cancer Patients Aged ≥55 Years in the SEER-Medicare Population

**DOI:** 10.1155/2008/931532

**Published:** 2008-08-27

**Authors:** Charles Kunos, Heidi Gibbons, Fiona Simpkins, Steven Waggoner

**Affiliations:** ^1^Department of Radiation Oncology, Case Comprehensive Cancer Center and University Hospitals of Cleveland Case Medical Center, Cleveland, OH 44106, USA; ^2^Department of Obstetrics and Gynecology, Division of Gynecologic Oncology, Case Comprehensive Cancer Center and University Hospitals of Cleveland Case Medical Center, Cleveland, OH 44106, USA; ^3^Department of Obstetrics and Gynecology, Division of Gynecologic Oncology, Cleveland Clinic, Cleveland, OH 44197, USA

## Abstract

Our study evaluated whether 1999 National Cancer Institute (NCI) chemoradiation guidelines for cervical cancer impacted treatment of women ≥55 years. We identified 385 women ≥55 years (median, 72 years) diagnosed with stage II-IVA cervical cancer between January, 1998 and December, 2002 in the United States Surveillance, Epidemiology, and End Results (SEER)-Medicare registries. Chemoradiation frequency tables were constructed for age, race, community setting, socioeconomic status, and comorbidity index. Of 385 women, 166 (43%) received chemoradiation as primary treatment. Prior to the 1999 NCI clinical alert, 5/43 (12%) in 1998 and 24/54 (44%) in 1999 received chemoradiation. The chemoradiation proportion was 41% (36/87) in 2000, 48% (51/107) in 2001, and 53% (50/94) in 2002 (trend, *P* < .01). Women ≥71 years had significantly lower odds of chemoradiation (*P* = .04). While SEER-Medicare data indicated an increasing trend for chemoradiation after the 1999 NCI clinical alert, chemoradiation was less frequent in elderly women with cervical cancer.

## 1. Introduction

Cervical cancer is the second most frequently diagnosed cancer among women worldwide, with nearly 80 percent arising among women in developing countries [1]. Despite effective screening modalities, nearly 40 percent of women in the United States present with locally advanced stage II-IVA cervical cancers extending beyond the cervix to invade vaginal or parametrial, pelvic sidewall, bladder, or rectal tissues [1].

In women with locally advanced cervical cancer, three randomized trials have shown concurrent pelvic radiation and platinum-based chemotherapy meaningfully contribute to improved disease-free survival [2–4]. These compelling clinical results prompted the National Cancer Institute (NCI) to issue a publicized national clinical alert in February, 1999 advising concurrent platinum-based chemotherapy and radiation for women with locally advanced cervical cancer [5].

According to the United States Surveillance, Epidemiology, and End-Results (SEER) cancer registries between January, 1998 and December, 2002, 35 percent of annual incident cervical cancer patients arose in women aged 55 years or older (≥55). However, women ≥55 years accounted for 55 percent of cervical cancer-related mortality during this time period. Widening disparity between cervical cancer incidence and mortality among women ≥55 years questions whether chemoradiation has been implemented as a national practice pattern in this patient population. Population-based studies are needed to evaluate the national impact of the 1999 NCI clinical alert advising chemoradiation for locally advanced cervical cancer. The merged SEER-Medicare dataset offers a population-based cohort of women ≥55 years longitudinally tracked over the course of cancer diagnosis, treatment, and follow-up [6]. The utility of SEER-Medicare data to identify chemotherapy administration has been validated [7]. This study determines the frequency of chemoradiation administration in women ≥55 years diagnosed with cervical cancer immediately before and after the 1999 NCI clinical alert using the population-based SEER-Medicare dataset. Secondary aims included identifying factors in women aged ≥55 years associated with decreased chemotherapy administration during radiation.

## 2. Methods

### 2.1. Data Source

Data were abstracted from the NCI-sponsored SEER-Medicare program using the SEER 11-registries plus Alaska 1998–2003 dataset (July, 2006 edition, [8]). Because SEER data contain no unique personal identifiers and thus are available for public use and research, approval by an ethics committee or informed consent is not necessary to perform our analyses. In general, SEER data cover 26 percent of the US population, and therefore, are considered representative of the whole US population [8]. The SEER program registries routinely collect data on patient demographics (i.e., age, race [Caucasian, African-American, Asian, Hispanic, or other], patient residence, and socioeconomic status [income per census tract]), primary tumor site (i.e., cervix), tumor morphology and stage at diagnosis, first course of treatment, and follow-up for vital status. The SEER program does not record chemotherapy administration.

To identify claims for chemotherapy administration [7], data were abstracted from three merged SEER-Medicare files: Medicare provider analysis and review (MEDPAR), carrier claims (NCH), and outpatient claims (OUTSAF). MEDPAR files record all calendar year part A short stay, long stay, and skilled nursing facility claims. NCH files contain calendar year Center for Medicare and Medicaid Services (CMS) physician/supplier part B claims. OUTSAF files list calendar year claims for hospital outpatient departments, rural health clinics, renal dialysis facilities, outpatient rehabilitation facilities, comprehensive outpatient rehabilitation facilities, and community mental health centers [9]. Procedure codes for chemotherapy administration made within 3 months after diagnosis of cervical cancer were ascertained [10].

### 2.2. Study Population

The study population consisted of all female patients, without previous or synchronous invasive cancers, who were diagnosed with stage II, III, or IVA cervical cancer between 1998 and 2002 (*n* = 3, 601). Stage I patients (*n* = 1, 363) were excluded because they are not reliably substaged (i.e., IB1 or IB2) in the SEER-Medicare database and the majority underwent definitive surgery (1,044 of 1,363 [76.6%]). Women with unknown stage (*n* = 357), stage 0 (*n* = 47), and stage IVB (*n* = 402) cervical cancers were excluded. To ensure that chemotherapy claims coincided with radiation and are not missed secondary to payment by private insurance (*n* = 431), included women must have had Medicare parts A and B coverage for the duration of their cancer care (*n* = 1, 001) [7]. For determination of chemoradiation administration, included women ≥55 years must have undergone radiation (external pelvic and brachytherapy implant) for primary treatment of their cervical cancer (*n* = 385).

### 2.3. Comorbidity Index

The Charlson comorbidity index is a summary measure of 19 comorbid conditions, each assigned a weighting factor according to its potential for influencing mortality [11]. Charlson comorbidity indices [11] were calculated by abstracting Medicare ICD-9-CM diagnosis and procedure codes relating to cardiovascular, cerebrovascular, hepatic, renal, diabetic, connective tissue, or neoplastic disease among inpatient and outpatient claims made for each woman one year prior to the diagnosis of cervical cancer [12–14]. Women diagnosed with chronic end-stage renal disease or listed in the SEER-Medicare dataset as having diagnosis codes for renal disease, renal failure, or renal insufficiency were considered women as having renal disease comorbidity [10].

### 2.4. Statistical Analysis

For this analysis, the day of diagnosis in SEER was arbitrarily assigned as the 15th of the month. Women were considered to have received concurrent chemoradiation therapy if there was at least one submitted billing claim for chemotherapy within a three-month period after diagnosis and radiation treatment. Chi-square tests for categorical variables and Student's *t*-tests for continuous variables were used to compare differences in demographic and tumor variables by chemoradiation administration. Adjusted odds ratios for chemoradiation were generated using multivariate logistic regression models sequentially controlling age, race, residence, socioeconomic status, comorbidity, tumor stage, and histology as defined in Table 1. A *P*-value *α* < 0.05 (two-sided) was used to determine statistical significance. Computer programing and statistical analyses were performed using SAS software version 9.1 (SAS Institute, Cary, NC).

## 3. Results

### 3.1. Patient Demographics

After exclusions, 385 women aged ≥55 years underwent pelvic radiation and intracavitary brachytherapy for primary treatment of cervical cancer between January, 1998 and December, 2002. Median age was 72 years (range 55–94 years). Table 1 presents patient demographic and tumor variables for the 385 women included in this analysis. The majority of cases were stage II squamous cell cancers diagnosed in Caucasian women who had minimal comorbidities and who resided in large metropolitan areas.

### 3.2. Concurrent Chemoradiation

Among women aged ≥55 years identified in the SEER-Medicare dataset, 166 of 385 (43%) received chemoradiation. Cisplatin chemotherapy was specifically claimed in 129 of 166 (78%) women. Before the 1999 NCI clinical alert, 30 percent of women aged ≥55 years (29 of 97 women) identified in the SEER-Medicare registry received at least one chemotherapy dose during radiation (see Table 2). In the year 2000 and after the 1999 NCI clinical alert, the proportion receiving chemoradiation was 41 percent of women aged ≥55 years (36 of 87 women). Afterwards, the proportion receiving chemoradiation was greater than 50 percent (see Table 2). Between 1998 and 2002, a significant increasing trend for chemoradiation administration was observed among women aged ≥55 years registered in the SEER-Medicare dataset (*P* < .01). The median number of chemotherapy claims during radiation was four (mean 4.2, standard deviation 1.9, Table 2). Among women treated with chemoradiation, 78 of 166 (47%) received five or more cycles of chemotherapy. Table 3 compares variables in univariate analyses evaluating patients who received concurrent chemotherapy and radiation compared to those who received radiation alone. The mean age of women receiving chemoradiation was significantly younger (Table 3, *P* < .001). The proportion of women residing in urban, less urban, and rural areas receiving chemoradiation was significantly higher than receiving radiation alone (20% *versus* 10%, *P* = .03). Chemotherapy use during radiation was not associated with race, socioeconomic status, tumor histology or stage, or comorbidity index. The proportion of women diagnosed with renal disease comorbidity was similar among chemoradiation (18/166, 11%) and radiation alone (21/219, 10%) treatment groups (*P* = .73).

Table 4 presents multivariate analyses for odds of receiving concurrent chemoradiation adjusting for patient demographic and tumor differences. Women aged 71 years or older had a significantly decreased odds of undergoing chemoradiation treatment as compared to women aged 55 to 70 years (*P* = .04). For women 71 years or older, the proportion of women with renal disease who received chemoradiation (9 of 95, 10%) and radiation alone (7 of 138, 5%) was similar (*P* = .19). In multivariate analyses, women residing in urban, less urban, and rural areas had significantly increased odds of undergoing chemoradiation treatment as compared to women residing in metropolitan areas (*P* < .001). The proportion of women 71 years or older in big metropolitan, metropolitan, and urban/less urban/rural areas was 42 percent (99 of 235), 35 percent (33 of 61), and 36 percent (20 of 56), respectively (*P* = .41). Race, socioeconomic status, comorbidity index, and tumor histology or stage were not significant confounders for chemoradiation treatment.

## 4. Discussion

The present data from the US population-based SEER-Medicare database indicate that the 1999 NCI clinical alert for platinum-based chemoradiation has had an impact on chemoradiation use for locally advanced stage II-IVA cervical cancers diagnosed in women ≥55 years. Eighteen months before the NCI clinical alert, less than 30 percent of women ≥55 years received chemoradiation. However, within 18 months of the NCI clinical alert, the proportion of women ≥55 years with locally advanced cervical cancer receiving chemoradiation significantly rose to 50 percent. Since chemoradiation use has steadily risen through the last SEER-Medicare cohort available for study (i.e., 2002), the recommendation has been followed but a substantial fraction of women ≥55 years still did not receive concurrent chemoradiation in 2002 three years after the NCI clinical alert.

Age is a risk factor for pelvic and distant relapse as well as death after treatment for cervical cancer. In multivariate analyses conducted by the Gynecologic Oncology Group evaluating radiation and chemotherapeutic radiation sensitizers [15], older patient age at diagnosis significantly conferred less cervical cancer recurrence or death. These findings led to the controversial suggestion that younger patient age at diagnosis was associated with high mortality; and yet, the inferior mortality outcomes attributable to younger patient age have vanished in the era of platinum-based chemoradiation as no contemporary Gynecologic Oncology Group or cooperative group study identifies age as a significant confounder upon survival outcomes [2–4]. While platinum-based chemoradiation appears to have decreased mortality in younger women, it remains unknown whether older women are deriving full survival benefit from chemoradiation treatment. Perceived and actual hematological, renal, gastrointestinal, and neural toxicities of platinum chemotherapy perhaps influence physicians to not consider coadministration of chemotherapy during radiation in older women. These data indicate that significantly fewer women older than 71 years who have locally advanced cervical cancer receive chemoradiation—even though the survival of these older patients who receive chemoradiation approaches that of younger patients who undergo chemoradiation treatment [2–4, 16]. This study is not the first to identify this age trend.

In a pattern of care study [17], it was reported that 63 percent of all women captured in this survey underwent concurrent chemotherapy and radiation in 1999, as compared to 26 percent in 1998. Twenty-five percent of women 60 years or older received chemoradiation as compared to 48 percent among women aged 40 years or less (*P* = .02) [17]. In an earlier analysis of the SEER-Medicare database controlling for racial and socioeconomic factors, increasing age was strongly associated (*P* < .0001) with receiving neither chemoradiation nor radiation treatment for cervical cancer [18]. However, both studies capture only those women treated through 1999, study periods ending when the 1999 NCI clinical alert were released. Thus, a true assessment of the impact of the NCI clinical alert is essentially unknown.

The present data are the first assessment of chemoradiation treatment after the 1999 NCI clinical alert for chemoradiation treatment of locally advanced cervical cancer in women aged ≥55 years. The US SEER-Medicare dataset is an NCI-sponsored cancer registry that reflects a wide range of race/ethnic, demographic, and socioeconomic diversity and that avails itself to cancer health service delivery analyses in women aged ≥55 years. The data presented herein reflect prospectively collected but not randomized practice and usage of chemoradiation. These data measure the impact of randomized clinical trials in cervical cancer, as popularized by the national 1999 NCI clinical alert, on clinical chemoradiation practices for the treatment of cervical cancer. Thus, these data conveniently sample a small proportion of the total number of women with locally advanced cervical cancer treated in the US each year. Consequently, analyses on these nonrandomly treated women can only be considered as an interpretive guide for changing trends in chemoradiation usage for locally advanced cervical cancer. Indeed, the present data suggest that women ≥55 years with locally advanced cervical cancer are increasingly likely to receive chemoradiation. Data also suggest that chemoradiation use has significantly lagged behind in women 71 years or older compared to women 55 to 70 years. Perhaps physicians applied the NCI recommendations to some groups of women more consistently than others.

In this study of the SEER-Medicare population, age emerges as an important factor influencing whether patients receive chemoradiation. While the NCI clinical alert has strongly endorsed use of chemoradiation for all patients with locally advanced cervical cancer, it is worth considering the causes of disparity in chemoradiation use among younger and older women. The exact regimen of chemotherapy administration, patient-specific chemotherapy dosage, and adverse effects of treatment cannot be abstracted reliably from the SEER-Medicare database. While reluctance of older patients to receive chemotherapy due to perceived toxicities, tolerability, or an apathetic attitude often are difficult to enumerate, physical limitations such as comorbid disease may be more reliably estimated in a population-based database that records these factors. In the current study, a high Charlson comorbidity index was not associated with more infrequent chemoradiation treatment. Comorbid renal disease, as identified by diagnosis codes for renal disease, renal failure, or renal insufficiency, also was not associated with infrequent chemoradiation treatment.

Demographic variables of race, socioeconomic status, and tumor variables of histology and clinical stage were not associated with more infrequent chemoradiation treatment, a reassuring finding of this study. However, a significant disparity in the proportion of women ≥55 years in metropolitan and nonmetropolitan residencies was observed in this study. Only 40 percent of the women residing in metropolitan areas as compared to 60 percent of women residing in urban, less urban, and rural residencies received chemoradiation during the entire 1998 to 2002 period under study (*P* = .03). Disparity in age cohorts among residences does not account for this difference as the proportion of women aged 71 years or older among big metropolitan, metropolitan, and urban/less urban/rural areas is similar (*P* = .41). The issues surrounding the more frequent use of radiation alone in metropolitan residencies are difficult to abstract but perhaps are related to unreported comorbid disease, patient refusal, and perceived toxicities/tolerability of the combined treatment regimen. The data cannot exclude the possibility that healthcare-system-related factors, such as access to care or differences in referrals for chemotherapy consultation, could explain the findings of the current study. The reasons why older women do not receive chemotherapy remain elusive, and perhaps, future population-based studies may provide more meaningful insight.

Our study is not without interpretative criticism. The population covered by SEER program representing 26 percent of the general population compares favorably to US census population data with regard to measures of poverty and education, but has a higher proportion of urban and foreign-born persons in the registry as compared to the general US population. Census population estimates for the year 2000 [19] suggest that 73 percent of women aged ≥55 years live in metropolitan areas as compared to 85 percent registered in the SEER-Medicare dataset. There may also be differences in the underlying racial distribution of the SEER-Medicare dataset compared to the US population comprising new incident cases of cervical cancer [20]. This SEER-Medicare dataset used to analyze the impact of the NCI clinical alert is limited to women aged ≥55 years that must have been enrolled in Medicare Part A and B coverage for the duration of their cervical cancer treatment. Thus, by limiting the dataset by an age criterion of ≥55 years, this analysis does not capture chemoradiation treatment in young women who comprise the majority of new incident cases in the US. Moreover, identification of chemoradiation in the SEER-Medicare population follows the Centers for Medicare and Medicaid Services' coverage policies relating to chemotherapy administration. Current Centers for Medicare and Medicaid Services policy permits fee-for-service reimbursement for intravenous chemotherapy [7], including cisplatin chemotherapy administration. As Medicare billing claims for chemotherapy administration are often coded within six months of initial cancer diagnosis [7], this analysis' three-month timeframe for coincident chemotherapy and radiation treatment in the SEER-Medicare dataset is reasonable. Women simultaneously enrolled by a private insurer were excluded as chemotherapy administration may have been paid by this entity rather than Medicare, and thus falsely skewing results toward treatment with radiation alone. Another limitation is that comorbid disease documentation in a population-based dataset is derived from claims-based administrative coding, which may generally be less complete than data obtained from direct medical chart review. Interpretative criticism also concerns the timeliness of the SEER-Medicare cohort under study, as these patients may be poorly reflective of modern chemoradiation practice; and yet, SEER data and Medicare data are infrequently merged. As such, the 1998–2002 SEER-Medicare patient population represents the most contemporary cohort in which to study concurrent SEER radiation and Medicare chemotherapy recorded administration.

The current analysis of 1998–2002 SEER-Medicare data indicates increased utilization of chemoradiation treatment since the 1999 NCI clinical alert strongly advocating for concurrent chemotherapy and radiation for locally advanced cervical cancer. Older age has been considered a barrier to chemotherapy. Between 1998 and 2002, the population-based SEER-Medicare registry identifies 43 percent of the women ≥55 years with locally advanced cervical cancer receiving chemoradiation despite published guidelines to the contrary. While our study is provocative, it is unable to definitively assess current national chemoradiation practice patterns. To further corroborate our findings, it would be interesting to analyze NCI-sponsored clinical trials enrolling women aged ≥55 years to determine if the number of chemotherapy cycles given during radiation impacts clinical outcome. Further analysis of national practice patterns from future US Surveillance, Epidemiology, and End-Results-Medicare mergers is warranted to identify contributing factors leading to the disparity in incidence and mortality among women ≥55 years with locally advanced cervical cancer.

## Figures and Tables

**Table 1:** Patient demographics (*n* = 385).

Age*	73.3 ± 0.37 (55–94 years)
Stage	
II	213 (55.3%)
III	163 (42.3%)
IVA	9 (2.3%)

Year of diagnosis	
1998	43 (11.2%)
1999	54 (14.0%)
2000	87 (22.6%)
2001	107 (27.8%)
2002	94 (24.4%)

Histology	
Squamous	289 (75.1%)
Adenocarcinoma	55 (14.3%)
Adenosquamous	13 (3.4%)
Other	28 (7.3%)

Race	
Caucasian	237 (61.6%)
African-American	74 (19.2%)
Asian	34 (8.8%)
Hispanic	25 (6.5%)
Other	15 (3.9%)

Location	
Big metropolitan (≥250,000)	235 (61.0%)
Metropolitan (20,000 to 249,999)	94 (24.4%)
Urban (2,500–19,999, adjacent metropolitan area)	17 (4.4%)
Less urban (2,500–19,999, not adjacent metro area)	32 (8.3%)
Rural (<2,500)	7 (1.8%)
Socioeconomic status (median income per census tract)*	$44,540 ±$1,125

Comorbidity index, Charlson	
0	274 (71.2%)
1	63 (16.4%)
2	28 (7.3%)
3+	20 (5.2%)

*Mean ±Standard Error of Mean.

**Table 2:** Concurrent chemoradiation by year of treatment and by number of claims (cycles) within ±3 months of radiation.

Year*	# Chemoradiation/Total # Women
1998	5/43 (11.6%)
1999	24/54 (44.4%)
2000	36/87 (41.4%)
2001	51/107 (47.7%)
2002	50/94 (53.2%)

Cycles	Total # Women
1	20
2 to 4	68
5 to 6	69
7 or more	9

*MH Chi-square = 15.83; *P* < .001 for trend.

**Table 3:** Univariate comparison of patients who received chemoradiation treatment versus those who received radiation treatment alone.

	Chemoradiation (*n* = 166)	RT alone (*n* = 219)	*P* value*
Age	71.7 ± 0.48	74.4 ± 0.53	<.001

Stage			.393
II	89 (53.6%)	124 (56.6%)	
III	74 (44.6%)	89 (40.6%)	
IVA	3 (1.8%)	6 (2.7%)	

Histology			.628
Squamous	129 (77.7%)	160 (73.1%)	
Adenocarcinoma	23 (13.8%)	32 (14.6%)	
Adenosquamous	4 (2.4%)	9 (4.1%)	
Other	10 (6.0%)	18 (8.2%)	

Race			.445
Caucasian	97 (58.4%)	140 (63.9%)	
African-American	34 (20.5%)	40 (18.3%)	
Hispanic	6 (3.6%)	9 (4.1%)	
Asian	14 (8.4%)	20 (9.1%)	
Other	15 (9.0%)	10 (4.6%)	

Location			.028
Big metropolitan (≥250,000)	88 (53.0%)	147 (67.1%)	
Metropolitan (20,000 to 249,999)	44 (26.5%)	50 (22.8%)	
Urban (2,500–19,999, adjacent metro area)	11 (6.6%)	6 (2.7%)	
Less urban (2,500–19,999, not adjacent metro area)	19 (11.4%)	13 (5.9%)	
Rural (<2,500)	4 (2.4%)	3 (1.4%)	

Socioeconomic status (median income per census tract)	$44,308 ±$1,512	$44,715 ±$1,614	.858

Comorbidity index, Charlson			.641
0	116 (69.9%)	158 (72.1%)	
1	31 (18.7%)	32 (14.6%)	
2	10 (6.0%)	18 (8.2%)	
3+	9 (5.4%)	11 (5.0%)	

*Student's *t*-test or chi-square for frequency data.

**Table 4:** Multivariate analysis for chemoradiation administration within 3 months after diagnosis in women with stages II-IVA cervical cancer from 1998 to 2002.

Patient and tumor characteristics	number of cases (*n* = 385)	Odds ratio* of chemoradiation	95% Confidence interval
Age			
55–70 years	152 (39.5%)	1	—
>71 years	233 (60.5%)	0.62	0.42–0.94

Stage			
II	213 (55.3%)	1	—
III	163 (42.3%)	0.96	0.63–1.47
IVA	9 (2.3%)	0.59	0.14–2.50

Histology			
Squamous	289 (75.1%)	1	—
Adenocarcinoma	55 (14.3%)	0.84	0.45–1.57
Adenosquamous	13 (3.4%)	0.53	0.16–1.78
Other	28 (7.3%)	0.61	0.26–1.43

Race			
Caucasian	237 (61.6%)	1	—
African-American	74 (19.2%)	1.22	0.72–2.06
Other	74 (19.2%)	1.28	0.75–2.18

Residence			
Big Metropolitan area	235 (61.0%)	1	—
Metropolitan area	94 (24.4%)	1.48	0.89–2.45
Urban, less urban, rural	56 (14.5%)	2.63	1.41–4.91

Socioeconomic status, median income			
<30,700	94 (24.4%)	1	—
30,701–39,425	93 (24.2%)	0.77	0.47–1.28
39,426–53,707	102 (26.5%)	0.72	0.43–1.20
>53,708	96 (24.9%)	1.18	0.70–1.98

Comorbidity index, Charlson			
0	274 (71.2%)	1	—
1	63 (16.4%)	1.09	0.61–1.94
2	28 (7.3%)	0.72	0.31–1.65
3+	20 (5.2%)	0.8	0.31–2.10

*Adjusted for variables listed in the tables.
